# Immunological pathogenesis and treatment progress of adenovirus pneumonia in children

**DOI:** 10.1186/s13052-024-01836-1

**Published:** 2025-01-09

**Authors:** Yaowen Liang, Jie Wei, Jianjun Shen, Zihao Liang, Xiuchang Ma, Yuchen Du, Wenxian Qian, Hui Dong, Ping Huang, Apeng Chen, Changhua Yi

**Affiliations:** 1 The Second Hospital of Nanjing, Affiliated Hospital to Nanjing University of Chinese Medicine, Nanjing, China; 2 Department of Chinese Medicine, The Second Hospital of Nanjing, Affiliated Hospital to Nanjing University of Chinese Medicine, Nanjing, China; 3 Department of Hepatology, The Second Hospital of Nanjing, Affiliated Hospital to Nanjing University of Chinese Medicine, Nanjing, China; 4 Children’s Hospital of Nanjing Medical University, Nanjing, China

**Keywords:** Human adenovirus, Pneumonia, Children, Immunomodulatory therapy

## Abstract

Human adenovirus is an infectious agent that causes respiratory infections in adults and children. It has been found that immunocompromised children are highly susceptible to this pathogen, as it can swiftly evolve into severe pneumonia with multiple sequelae. Due to the lack of immunity in children, the body’s response mechanisms to innate and acquired immunity are specialized. We first examined the infection classification and clinical characteristics associated with adenovirus in children. Subsequently, we explored the in-depth understanding of the pathogenic mechanism of adenovirus pneumonia in children, focusing on immunological and cellular biological aspects. Adenovirus infection in children can disrupt the balance of the innate immune response, inducing immune cells to secrete an abundance of pro-inflammatory cytokines. This cascade results in a cytokine storm, which triggers an inflammatory response and causes lung tissue damage. As a result, the infection may progress to a severe state, potentially leading to multi-organ failure. Immunocompromised children exhibit impaired immune cell numbers and functions, which affects both the secretion of antibodies to humoral immunity and the immune response of cellular immunity to adenovirus. Lastly, we reviewed the progress in treating adenovirus pneumonia in children. There are many treatments for adenovirus pneumonia in children, which must be personalized based on a thorough assessment to optimize treatment outcomes. Recent advancements in pharmaceutical development have provided new treatment options for children. Immunomodulatory therapy can reduce inflammation in children, while adjuvant therapy can improve respiratory function; however, it can also lead to complications. Further, co-infections increased the complexity of diagnosis and treatment, necessitating dynamic adjustments to treatment regimens. This review could serve as the basis for identifying potential therapeutic approaches to alleviate the symptoms associated with adenovirus infections in children.

Adenovirus is a non-enveloped virus with double-stranded DNA that is a major infectious cause of respiratory disease in children [1]. Human adenovirus (HAdV) infection usually results in respiratory or gastrointestinal symptoms in humans and is self-limiting but can be fatal in immunocompromised patients or newborns [2]. Adenovirus pneumonia (AVP) poses a significant risk to children’s health, comprising 4–10% of respiratory infections and cases of viral pneumonia in children [3]. AVP can occur at any age; however, infants below the age of 6 months are particularly susceptible to the infection. As infants grow older, their levels of maternal antibodies decrease, leaving their respiratory and immune systems vulnerable. This makes them more susceptible to developing adenoviral bronchiolitis and experiencing various complications throughout their body. These complications can lead to acute lung injury, residual lung injury, severe adenovirus pneumonia (SAP), and even fatal hypoxemia and acute respiratory distress syndrome in severe cases [4–6].

The early clinical manifestations of AVP in children are not specific. The clinical manifestations are unclear, usually with prolonged fever and cough. The disease progresses rapidly, and the lung lesions progress from nodular or patchy consolidation to significant consolidation within 1–2 days, with high mortality and poor prognosis, and there is no HAdV vaccine for children [7, 8]. And because the subgenus HAdV-B associated with severe pneumonia in children cannot infect rodents, it is impossible to establish a typical pneumonia model, and it is difficult to find a specific treatment [9]. A study has shown that the number of HAdV infection cases has decreased in recent years, but the evolutionary characteristics and severity of HAdV have not changed, and the fatality rate is higher than that of other studied viruses [7]. The pathogenesis of adenovirus infection in critically ill children is associated with lymphopenia and inflammatory response disorders, but the pathogenesis of AVP has not been fully elucidated, so it is particularly important to study the immunological pathogenesis of HAdV infection in children.

## Etiology of HAdV

The structure of HAdV has been determined at atomic resolution by cryo-EM [10]. The HAdV genome is about 36 kb in length and about 90 nm in diameter and consists of a protein capsid with a viral core inside. The capsid consists of 252 envelopes with an icosahedral symmetric structure. Each face consists of 12 pseudo-hexagonal hexons, and the tip consists of 12 pentons connected to one or more non-covalently bound fiber spikes [11].

HAdVs are based on nucleotide sequence characteristics, protein immunogenicity, and serological definition of the HAdV genome and are members of the family Adenoviridae. There are currently 7 subgenera (A-G) and 118 genotypes (Human Adenovirus Working Group, Nov. 2023 update, http://hadvwg.gmu.edu). About 67 serotypes are known to be pathogenic to humans. HAdV serotypes have different degrees of infectivity and virulence in children and adults, and their infections can lead to various diseases [12] (Table 1), as subgenera A, F, and G are associated with gastrointestinal infections; subgenera C and D are related to respiratory and eye infections; and subgenus B is associated with respiratory, renal and urinary tract infections. HAdV1, 2, 3, 4, 5, 6, 7, 11, 14, 21, 34 and 35 are mainly associated with respiratory disease [13, 14]. The newly identified 14PI type, recombinant 55 type, and HAdV types 3, 4, 7, and 14 are the main types of acute respiratory tract infection (ARTI) in children, often causing severe respiratory infections [15]. Among them, HAdV-3 and HAdV-7 are essential pathogens causing respiratory infections in children, and HAdV-7 has a stronger replication ability, which can intensify cytokine response, and patients have multiple tachypnea, lung lobar infiltration, and increased proportion of pleural effusion, resulting in higher severity of respiratory inflammatory reactions, PICU occupancy rate, invasive mechanical ventilation, and mortality are higher than those of other types [16–18].

Xu et al. found in a differentiated study of different serotypes of HAdV that HAdV-B55 can cause co-infection with pathogens such as Human parainfluenza virus and Influenza virus in hospitalized children, and the severity of the disease may be greater than that of HAdV-B7 [19]. In histopathological studies of SAP deaths, exudative diffuse alveolar injury and inflammatory cell infiltrating bronchial congestion were seen, and the lungs had necrotizing karyorrhectic debris [5]. These phenomena and laboratory test data indicate the irreversibility of severe AVP disease progression, which is a serious challenge for physicians.

**Table 1:** Human adenoviruses-associated diseases. HAdV species have various infectivity and virulence in children and adults, and their infection can lead to different diseases. Among them, HAdV-B and -C are the main types of SAP in children

Subgenera	Serotype	Associated disease (primary)	References
A	12,18,31,61	Meningoencephalitis, pneumonia, cryptic enteric infection	[20–22]
B	3,7,11,14,16,21,34,35,50,55	ARTI, ARDS, CAP, URTI, pneumonia, tonsillitis, bronchitis, epilepsy, high-grade fever, pharyngoconjunctival fever, hemorrhagic cystitis, meningoencephalitis, bronchiolitis	[13, 14, 16–19, 21, 23–26]
C	1,2,5,6,57	ARTI, ARDS, pneumonia, epilepsy, high-grade fever, Sepsis, meningitis, Gastroenteritis, Sudden infant death syndrome	[13, 14, 16–18, 24, 25, 27, 28]
D	8,9,37	Viral conjunctivitis, meningoencephalitis	[21–23]
E	4	ARTI, pneumonia, conjunctivitis	[18, 22, 24]
F	40,41	Diarrhea, acute gastroenteritis	[28–30]
G	52	acute gastroenteritis	[21]

## Innate immune system

Host responses to HAdV infection involve innate and adaptive immunity. Innate immunity is mainly driven by virion-associated molecular patterns (VAMPs), which control the immediate immune response of pre-stored factors to viral infection, followed by early inducible immune response recognition of VAMPs, initiation of inflammatory response, rapid detection and elimination of multiple pathogens. There are many ways in which hosts have antiviral effects on the innate immune system, and this article mainly describes the local defense of defensins, the type I interferon response, the antibody complement action, the innate immune responses, and the proinflammatory responses due to the release of cytokines (CK).

### Local defensive effects of defensins

Antimicrobial peptides are basic polypeptides with biological activity and antibacterial activity induced by organisms, which are widely expressed in various animals and have the immune effect of directly killing pathogens in the rapid innate immune response generated by pathogen invasion. The antimicrobial peptides in the human body are mainly defensins, single-chain strong cationic polypeptides of 29–35 amino acid residues, widely expressed in the human body as α-defensins and β-defensins. α-Defensins are mainly secreted by small intestinal Paneth cells and neutrophils, of which intestinal eosinophils and respiratory epithelial cells secrete human defensins 5 (HD-5) and HD-6. Β-defensins are produced by the skin and mucosal epithelial tissues [31, 32]. A study has shown that defensins have direct anti-HAdV activity, which can induce the secretomotor of the IL-6 and IL-8, thereby enhancing the antiviral response to HAdV infection [33]. However, the role of defensins on Ad vectors is still unclear, and the specific antiviral mechanism needs to be elucidated.

### Type I interferon- immune response

**Fig. 1:**
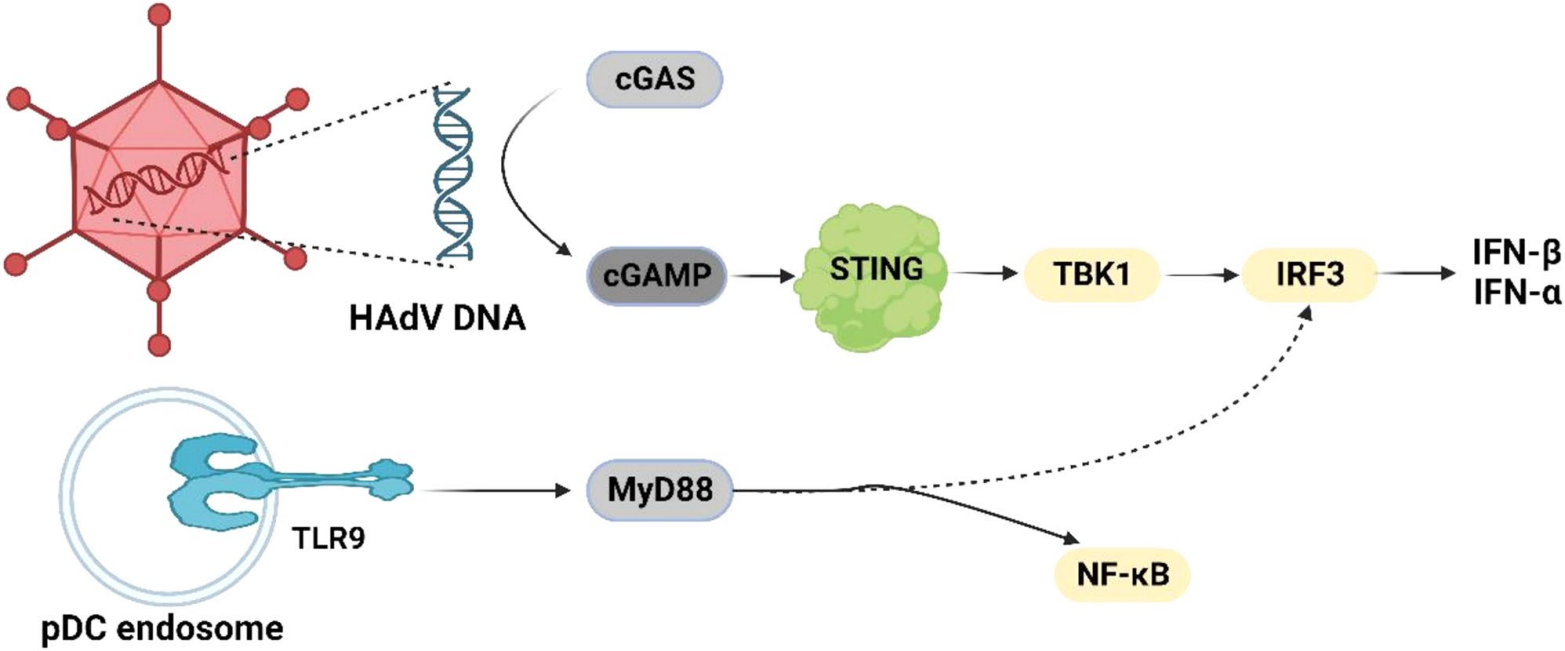
Cytoplasmic recognition of HAdV DNA occurs through cGAS sensing. cGAS senses DNA and synthesizes cGAMP. Thereafter, cGAMP binds to the adaptor protein STING, triggering the activation of TBK1 and IRF3, initiating the production of type I IFN, specifically IFN-β. The TLR/MyD88 signaling pathway activation occurs during HAdV infection and regulates NF-κB and MAPK kinetics. Created by http://www.Biorender.com

IFNs are a class of species-specific glycoproteins that can affect the cellular immune response. They are classified as IFN-α, IFN-β, IFN-γ, and IFN-λ based on their source and structure. Type I IFN, specifically IFN-α and IFN-β, is produced by infected viral cells. They are responsible for stimulating the utterance of antiviral genes in the pericytes, mediating the activation of the antiviral status of uninfected cells around the infection site, and it is involved in the intrinsic immune elimination of acute viral infection. The induction of HAdV by type I IFN involves two mechanisms: TLR-dependent and TLR-independent pathways [34]. (a) TLR-dependent pathway, in non-ex vivo isolated plasmacytoid dendritic cells (pDCs), the induction of IFN-α is activated by the cytoplasmic recognition of DNA and in a path of MyD88-independent, while the high -level production of IFN-α in pDCs is associated with high constitutive expression of interferon regulator factor-7 [35, 36]; (b) TLR-independent pathway, in conventional dendritic cells and macrophages, the transcription factor IRF-3 is stimulated by cytosolic DNA, triggering IFN-α and proinflammatory responses [37]. The guidance of HAdV vectors by IFN-β involves viral entrance, endosomal release, and exposing cytoplasmic HAdV DNA. TLR/MyD88 signaling is essential for the initiation of innate immunity. MyD88 signaling is activated by HAdV infection, adjustable nf-kappa B and MAPK dynamics, and induces the expression of CK [38]. The DNA sensor circular GMP-AMP synthetase (cGAS) facilitates cellular detection of HAdV DNA by identifying cytoplasmic DNA and synthesizing circular GMP-AMP (cGAMP). cGAMP can bind with high affinity to the adaptor protein STING, activating TBK1 and IRF3, activating IFN-β, and producing type I IFN reactions [39, 40]. However, specific expression of effector proteins and sensors can disrupt the signaling cascade from HAdV, resulting in unstable type I IFN reactions [41] (Fig. 1).

Type I IFN effectively prevents the replication of viruses by inhibiting the synthesis of viral structures, resulting in a direct antiviral state. Furthermore, type I IFN activity demonstrates direct antiviral effects and contributes to a range of immunomodulatory actions. Direct signaling of NK cells by type I interferon can promote NK cell activation and antigen presentation and enhance the role of both antigen-specific T cells and B cells. IFN-γ is called type II IFN and is associated with adaptive immunity. IFN-γ activation occurs through direct initiation of gene transcription from antigen stimulation by Th1-differentiated CD4 + T cells and NK cells. IFN-γ belongs to the macrophage activating factor (MAF), which can directly activate macrophages and can also enhance macrophage-killing capacity through T-cell action. Additionally, it plays a crucial role in class switching of IgG and IgE and cell expression of MHC [42].

### Antibody-complement action

After infection with HAdV, marginal B cells and some innate B cells secrete some IgA antibodies in the early stage and mainly secrete IgG and IgM antibodies. Against HAdV viral proteins and other host-specific epitopes, early antibodies (such as IgM) produce antiviral effects and immunostimulatory potential in vivo, effectively preventing lethal infections [43]. In newborns, natural antibody-secreting cells secrete natural IgM. The production of natural IgM antibodies does not require contact with antigens. Natural IgM is the first part of the defense against pathogen invasion and an important component of innate immunity [44]. Antibody-complement action is described in this part, and antibody neutralization is mainly an adaptive immunity, which we will describe in the next part.

**Fig. 2:**
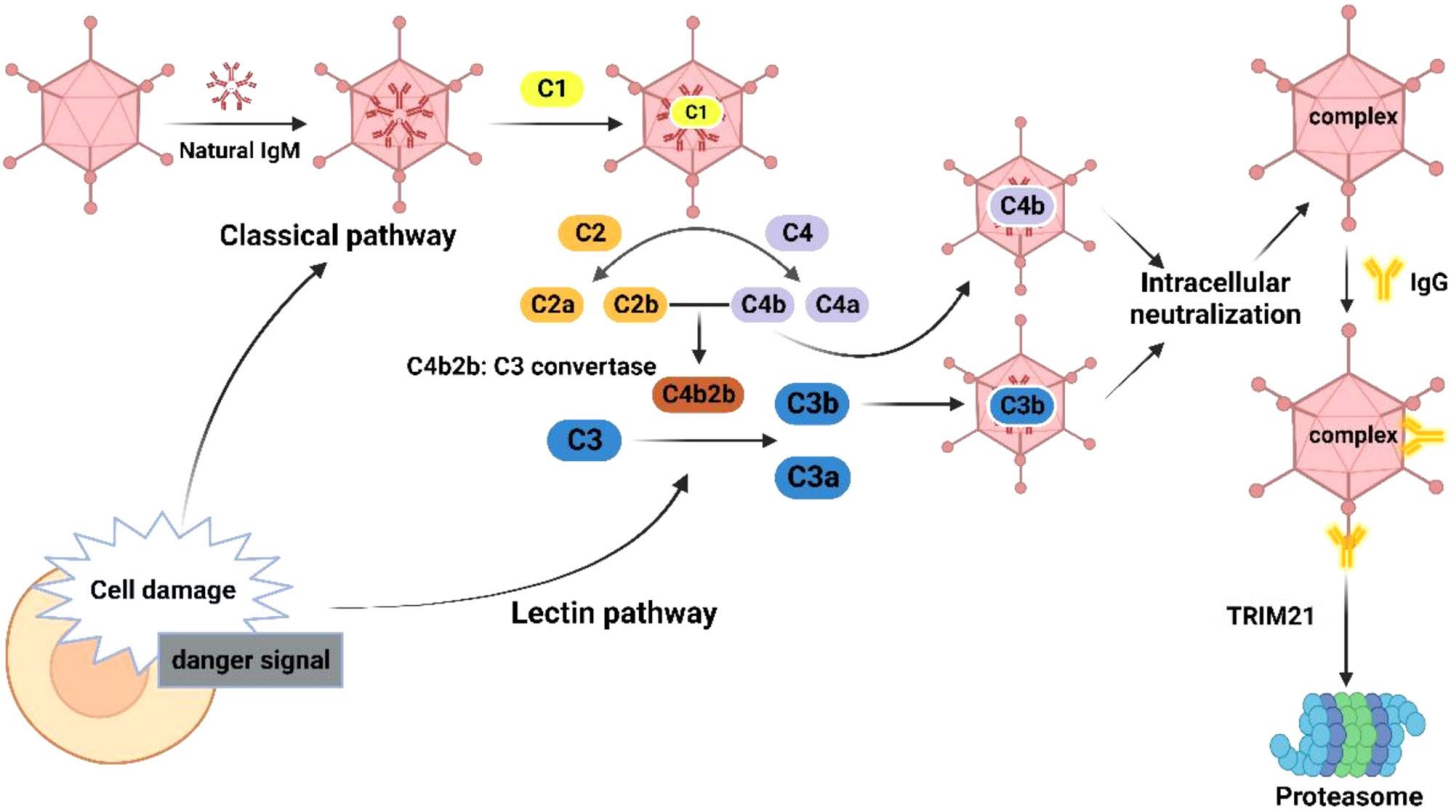
Complement activation pathways and antibody neutralization effects of HAdV infection. Left part: IgM triggers the activation of the complement system via the classical pathway. When IgM attaches to HAdV, it causes conformational changes that lead to the binding of complement protein C1q to antibodies. Once bound, C1 cleaves complement proteins C2 and C4. As a result, C4b and C2b combine to form C3 invertase, known as C4b2b. The C4b2b complex then cleaves C3 into C3a and C3b. Right part: The virus-antibody complex recruits TRIM21 to bind to IgG. This binding allows for the targeted transportation of the virus-antibody complex to the proteasome through E3 activity. Created by http://www.Biorender.com

Complement, a soluble molecule in innate immunity, acts as a pattern recognition element. The complement system consists of proteolytic cascade reactions, which can collectively detect and label pathogens, contributing significantly to the body’s immune defense by triggering various defensive actions. The complement cascade consists of two tightly coupled phases, with the C3 invertase activation phase encompassing the following pathways: the classical pathway, the lectin pathway, and the bypass pathway. The classical pathway is activated by antibodies (IgM and IgG) to recognize the pathogens and antibody-antigen complexes. On the other hand, the lectin pathway is triggered by the presence of mannose-binding lectin (MBL), which activates MBL-serine protease. The bypass pathway directly activates C3 through the involvement of factor B, factor D, and properdin (P) [45, 46]. The classical pathway is uniquely possessed by IgG& IgM, and its conformation changes after natural IgM binds to HAdV, inducing the binding of complement protein C1q to antibodies. After binding to the antibody, the activated C1 is cleaved into complement proteins C2 and C4, and two large fragments of the activated protein, C4b and C2b, are deposited on the cell surface to form the C3 invertase C4b2b; C4b2b then cleaves C3 into C3a and C3b [47, 48]. Complement action can inhibit HAdV binding to cells, and C3a can mediate inflammatory responses. C3b and C4b can covalently bind to HAdV, inhibiting its function within cells [49, 50] (Fig. 2).

### Innate immune response and cellular inflammation due to cytokine release

CK is a class of small molecule peptides or proteins composed of a variety of immune cells. The respiratory epithelial cells of hosts are targeted and infected by HAdV. The HAdV is released from the cell and interacts with the surface receptors on the target cell, initiating a downstream signaling cascade. This stimulates the activation of genes responsible for producing proinflammatory CK. Subsequently, the innate immune system releases numerous inflammatory mediators, leading to damage in normal cells and tissue inflammation and ultimately inducing severe lung infection [51]. Chemokine is a small molecular weight CK that fulfills a key function in the activation of lymphocytes and is secreted by chemotactic inflammatory cells through a concentration gradient. Non-chemokines exhibit indirect chemotactic effects and are essential to the different inflammatory and immune responses within the body. It has been reported that severe HAdV infection is associated with significant imbalances in inflammatory cell and cytokine responses, including TNF-α, IL-1, IL-6, IL-8, and IFN [52]. Immune system conditions can also be reflected by the levels of CK in the peripheral blood. Patients with AVP show elevated levels of IL-6, IL-8, IL-10, IFN-γ, and IL-22, but as their condition improves, IL-10 and IFN-γ levels decrease [53, 54].

TNF-α can stimulate systemic acute-phase responses in the host and mediates the acute inflammatory response. TNF-α is rapidly produced by macrophages and DC cells induced by PAMP or VAMP and induces apoptosis of tumor cells and other infected cells by participating in inflammatory and immune responses. TNF-α can induce C-X-C chemokine generation in type II epithelial cells and can induce colony-stimulating factor expression in vascular endothelial cells. This activation leads to neutrophil chemoattraction, release of additional inflammatory factors, and synergy with other CK, exacerbating viral infections and systemic complications [55, 56].

IL-1, secreted by alveolar macrophages, is the main inducer of the synthesis of IL-6 and IL-8, which is mainly involved in the acute-phase responses of HAdV infection, promoting T cell proliferation and enhancing the cytolytic capacity of CD8^+^ T cells [57]. IL-6 fulfills a function in the acute phase response to infection and, to a certain extent, can increase vascular permeability, impair organ functionality, and contribute to cytokine storms. It holds significance in viral infections by promoting immune defense against infected cells and contributing to tissue damage [8, 24]. It has been reported that IL-6 can also function as a hepatocyte-stimulating factor to promote the production of multiple acute-phase reaction proteins induced by the liver [58].

IL-8 plays a crucial role in controlling inflammation by attaching to the receptors CXCR-1 and CXCR-2 to promote neutrophil chemotaxis in the lung. Serum IL-8 levels have been observed to be significantly correlated with clinical prognosis in children with SAP, with elevated levels in children with ARDS and septic shock, and the highest levels in critically ill children [59]. Interestingly, another study found that HAdV-7 could increase the expression and release of IL-8, leading to severe infections in the lower respiratory tract [60]. A death case in a child infected by HAdV reported HAdV-7 expression in the nervous system, resulting in two persistent epileptic and systemic inflammatory responses. The reason is related to a pathogenic mutation of encoding vascular endothelial growth factor receptor 2 (VEGFR-2), of which the mutation and inflammatory response can double disrupt the selectivity and integrity of the blood-brain barrier, resulting in the viral invasion of the CNS. Still, the specific mechanism is not clear [61].

## Adaptive immune systems

The primary focus of adaptive immunity pertains to the deletion expression of derived genes in the early generation of HAdV vectors (E1 deletion HAdV). Due to the somatic recombination of recipient genes, the various cells of adaptive immunity (such as APC, T cells, and B cells) can specifically recognize the specific pathogens. Adaptive immunity consists of two components: Adaptive immunity consists of two components: cellular immunity mediated by T cells and humoral immunity mediated by B cells utilizing antibodies to carry out effector functions.

### Humoral immunity

After infection with HAdV, Children with the low age of the month produce high-affinity IgG antibodies. Antibody neutralization mainly refers to forming antigen-antibody complexes between antibodies and antigens through the Fab segment. This can facilitate the phagocytes to engulf the antigen effectively, and this effect is limited to the outside of the cell. The intracellular neutralization response of HAdV is primarily recognized and mediated by a tripartite motif containing 21 (TRIM21) (Fig. 2), a broadly characterized cell internal Fc receptor [62]. Virus-antibody complexes recruit TRIM21 to the Fc domain of IgG, which then catalyzes ubiquitin chain generation. This complex then interacts with the host AAA-ATPase-VCP and delivers the virus-antibody complex to the proteasome through targeted E3 activity [44, 63].

Consequently, the virion is rapidly degraded before encoding translation, thereby blocking infection. This process is known as intracellular antibody neutralization response. As infants develop, the antibodies obtained from their mother’s body decline. By the time they reach the age of five, approximately half of the children have acquired neutralizing antibodies to HAdV through either symptomless or mildly symptomatic infections.

As infants mature, they receive decreasing maternal antibodies, reaching their lowest point between 11 months and one year. Furthermore, nearly half of children by age 5 acquire neutralizing antibodies of HAdV through asymptomatic or mild-symptomatic infection. In the titer test of neutralizing antibodies to the different HAdV serotypes, it was found that the antibody levels of HAdV-7 and HAdV-55 in children were consistently low [64], indicating a lack of herd immunity against these HAdV serotypes. As a result, HAdV-7 can lead to severe infections among children, whereas HAdV-55, although causing fewer infections, is associated with higher disease severity than other serotypes.

### Cellular immunity

**Fig. 3:**
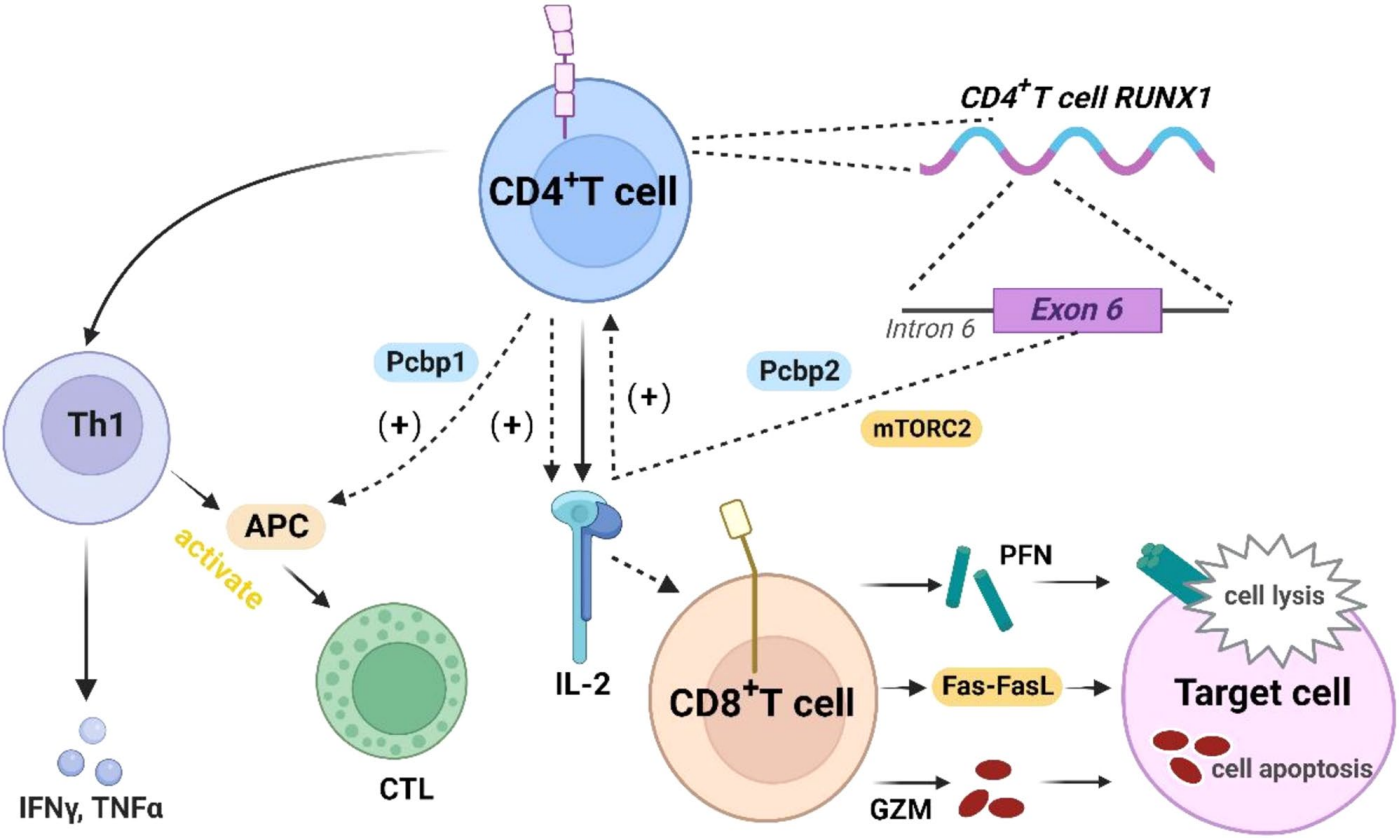
T-cells mediated adaptive immune responses. CD4^+^ T cells trigger the activation of APC by secreting Th1 to initiate CTL and secrete IL-2. Pcbps1 promotes CD4 + T to generate proinflammatory CK. Splicing RUNX1 exon 6 in PCBPS2 regulates IL-2 signal transduction through interaction with mTORC2, facilitating CD4^+^ T cell activation. Two molecular pathways of CD8^+^ T cells can induce apoptosis in infected cells: the granule exocytosis pathway and the Fas-FasL pathway. Created by http://www.Biorender.com

CD4⁺ T cells play an important role in combating adenovirus by releasing CK, including IL-2 and IFN-γ, which generate antiviral activity and promote B-cell antibody production [65]. Janssen et al. [66] found that for Th-independent antigens (such as viruses), CD4^+^ T can activate the ability of antigen-presenting cell (APCs) to initiate CTL by secreting Th1 cells. Th1-CD4⁺ T cells are arguably the important cells that activate macrophages and NK cells, thereby increasing HAdV clearance efficiency. However, the expansion of secondary CTL is completely dependent on Th cells during the priming process. Poly(C)-binding proteins (PCBPs/hnRNPEs) are a diverse family of RBPs consisting of Pcbps1 and Pcbps2. Pcbps1 can effectively promote CD4^+^ T to generate proinflammatory CK by the way of interacting with intracellular Fe inside of cells and stabilization of mRNA, preventing the conversion of effector T cells into regulatory T cells [67]. On the other hand, Pcbps2 functions as a splicing factor that can regulate the splicing of RUNX1 exon 6 in CD4^+^ T cells. It also promotes the activation of CD4^+^ T cells by regulating the co-stimulation pathway or IL-2 signaling via interaction with the intracellular signaling complex mTORC2, thereby maintaining the dynamic stability of the number of peripheral CD4^+^ T cells [68, 69]. (Fig. 3)

Furthermore, CD4⁺ T cells help maintain the balance of the immune system and prevent excessive inflammatory responses and lung tissue damage. Immunocompromised children experience reduced humoral immunity due to impaired accessory function of CD4⁺ T cells [70]. In addition, the reduced activity of Th1 and Th17 CD4⁺ T cells result in a decrease in CK production and CD8⁺ T cell number, as well as a weakening of the body’s immune function [71].

Regulatory T cells (Tregs) are a subset of the CD4 + T cell family that exert anti-inflammatory effects and promote damaged tissue repair through various mechanisms. These Tregs maintain the immune balance in local tissues and play a significant role in lung tissue recovery, inflammation regression, and injury repair [72]. In severe pneumonia, the body releases high levels of pro-inflammatory CK, triggering a “cytokine storm.” Also, a large amount of reactive oxygen species and proteases are released by the excessive activation of neutrophils, culminating in acute damage to lung tissue and the development of acute respiratory distress syndrome. Tregs mitigate tissue damage by inhibiting the pro-inflammatory response of macrophages through the release of anti-inflammatory CK such as IL-10 and TGF-β. In addition, they also reduce the expression of neutrophil chemokine CXCL8 and the inflammatory response. Tregs can utilize immune checkpoint molecules such as CTLA-4 and PD-1 to suppress the co-inhibitory molecule TIGIT, which helps in the prevention of excessive activation of effector T cells (such as Th1 and Th17) and reduce pro-inflammatory signaling in the lungs [73]. Further, Tregs can suppress APCs activity in local tissues, consequently indirectly inhibiting the prolonged activation of effector T cells [74]. The dysregulation of the TH17/Treg cell ratio serves as an important marker of inflammatory response in acute lung injury. Hence, restoring the balance of the TH17/Treg cell ratio has become a promising way to treat lung injury caused by severe pneumonia [75].

As AVP progresses to severe disease, heme activates Toll-like receptor-4, exerting pro-inflammatory effects. Tregs can induce histiocytes to produce Heme oxygenase- 1 (HO-1), which breaks down heme and produces anti-inflammatory molecules such as iron and biliverdin. This process reduces inflammatory responses, protects endothelial cells, and promotes the repair of locally damaged tissues [76, 77]. However, in severely ill children experiencing cytokine storm and immune disorder, the effects of HO-1 are relatively weak. Tregs exert a protective role by curbing excessive immune response, and this inhibition may lead to induce local immunosuppression during the acute phase of viral infection [78]. Although this mechanism proves detrimental in the acute phase, it prevents further damage to lung tissue from excessive immune response during the recovery from infection [79]. Research into Tregs immunotherapy for severe viral infections is still in its nascent stages. Studies have yet to fully elucidate the precise regulation of the activity of Tregs necessary to achieve effective anti-inflammatory properties. Additionally, the safety, efficacy, and duration of treatment of Tregs remain unresolved, but this approach provides a potential direction for the treatment of children with SAP.

CD8^+^ T cells specific to HAdV exhibit in-effect sample status by producing an effect in response to a stimulus. Moreover, these CD8^+^ T demonstrate continuous effector/memory-like characteristics through multiple responses. CD8^+^ T cells present virus-specific peptides to specifically recognize the various infected cells by MHC class I molecules and TCR. They can also induce apoptosis of infected cells through two different molecular pathways [80]: (a) Granule exocytosis pathway, which utilizes perforin (PFN) to deliver granzyme (GZM) into the target cells; (b) Fas-FasL pathway, upregulation of FasL can cause CD95 aggregation to induce apoptosis and produce a variety of proinflammatory CK [81, 82]. CD8^+^ T cells can rapidly upregulate perforin, which can be transported to immune synapses and induce enhanced cytotoxicity. The upregulation and degranulation of perforin are the potential co-effects of CTL activation [83]. Continuous generation and targeted loss of new perforin-induced CD8^+^ T cells enable them to identify and eliminate additional targets even after initial exhaustion, with new perforin being more likely to accumulate in CD8^+^ T cells that express T-bet, the principal controller of the CD8^+^ T cell reaction and an inhibitor of IL-2 transcription [84, 85]. Moreover, due to the high degree of sequence homology between ADVs of different serotypes, HAdV-specific T cell fractions often cross-react and can be stimulated by periodic infections of the various serotypes.

The immune response is further augmented by specific cell lysis mediated by CD8^+^ T cells. In AVP patients, killing target cells by CD8^+^ T cells can activate macrophage activation syndrome, resulting in immune system complications and tissue damage. A higher number of CD8 + T cells in children with acute AVP causes increasing CK levels like IFN-γ, suggesting that these T cells fulfill an important function with early virus eradication. Because HAdV can cause considerable damage to T cells and suppress the cellular immune function of patients by depleting immune cells, the proportion of CD4^+^ T/CD8^+^ T cells in critically ill children with immunodeficiency is significantly reduced. Immunocompromised children exhibit reduced CD8⁺T cell activity due to thymus deficiency, impaired peripheral immune organ function, or inadequate CD4⁺ T cells [86]. Memory CD8⁺ T cells are also essential for rapid viral recognition and increased immunity to pathogens. These cells in the lungs show high expression of integrin CD103 and chemokine receptor 6 (CXCR-6), which enhance its ability to reside in lung tissues and airway tissues, thereby preventing secondary infection of the virus [87, 88]. The insufficient production of immune memory in immunocompromised children results in a lack of effective memory CD8⁺ T cells in the body, and this deficiency in memory cells increases the risk of recurrent adenovirus infection.

During normal immune responses, CD8⁺ T cells release CK to enhance the antiviral microenvironment. However, in immunocompromised children, the production of key CK is reduced. This decrease affects the activity of immune cells such as macrophages and NK cells, reducing the body’s immune function. Consequently, the body becomes more susceptible to viral infection, increasing the risk of severe HAdV infection.

## Progress in treatment

Currently, the primary treatment approaches for children with AVP include conventional care, antiviral medications, and immunomodulatory therapy. Clinicians must carefully evaluate the child’s symptoms, laboratory results, imaging findings, and other information to formulate an appropriate treatment plan. Children with mild HAdV infection typically exhibit mild clinical signs and can recover without intervention after 1 ~ 2 weeks of conventional symptomatic treatment, generally leading to a good prognosis.

### Antiviral therapy

Due to the rapid and self-replicating nature of HAdV through the nucleus, there are no targeted antiviral medications for AVP in pediatric patients. Instead, broad-spectrum antiviral drugs such as cidofovir and ribavirin are commonly used [89]. These drugs indirectly reduce inflammation by suppressing viral replication, reducing viral load, and minimizing further damage to the body. While they do not directly reduce inflammation, these antivirals can slow the progression of inflammation by controlling the underlying infection [90].

Bisphosphonate, the main active substance in cidofovir, can inhibit the activity of HAdV, thereby hindering viral proliferation. Its antiviral activity has been proven through both in vitro and in vivo experiments. Cidofovir therapy has been demonstrated to be effective in severe pneumonia or ARDS caused by HAdV infection. Although cidofovir has been used for HAdV infection in children with organ and hematopoietic stem cell transplantation, substantial evidence supporting its effectiveness in immunocompetent children with SAP remains limited [91]. However, the nephrotoxicity and myelosuppression of cidofovir are still the most significant limitations in its clinical application [92]. Careful monitoring of creatinine, urea nitrogen, and other indicators is crucial during treatment, and further prospective experiments are needed to verify its efficacy. Brincidofovir, a novel cidofovir prodrug, demonstrates high bioavailability, low nephrotoxicity, and bone marrow toxicity. This formulation has been successfully applied to the prophylactic treatment of HAdV infection among children and adults receiving hematopoietic stem cell transplants. In addition, NPP-669, another prodrug of cidofovir, was more effective against adenovirus than Brincidofovir in animal experiments [93].

In vitro experiments have demonstrated that ribavirin exhibits effective antiviral properties against certain HAdV strains, but its efficacy is less pronounced in vivo experiments [94]. Clinical research indicates that ribavirin requires a plasma drug concentration higher than the standard dose to inhibit HAdV effectively. Consequently, its use is limited to experimental treatments for severely ill children. Studies have indicated that intravenous ribavirin with nebulized INFα1b shows a promising effect in the treatment and recovery of children with SAP, potentially offering a novel therapeutic method for future interventions [95]. After therapy, such as antiviral drugs and extracorporeal membrane oxygenation (ECMO), the immune system and immune system abnormalities in children entering the convalescent phase are rectified and stabilized. As a result, the ratio of CD4 + T cells to CD8 + T cells in their bloodstream substantially rises. This increase is a reliable indicator for assessing SAP severity and treatment efficacy.

### Immunomodulatory therapy

The severity of HAdV infection is influenced by both the pathogenicity of the virus and the immune response status of the child. Once the virus invades the body, it initiates an immune response and interacts with cellular receptors, resulting in the release of a variety of inflammatory mediators that are key contributors to the impairment of various organ functions. Currently, the commonly used immunomodulatory approach for pediatric HAdV infections involves intravenous immunoglobulin therapy [96].

Intravenous immunoglobulin (IVIG) functions to neutralize inflammatory factors and effectively inactivates them. The entry of numerous external antibodies can make the body produce a transient immune protection state. This process disrupts the intercellular interactions to recognize and clear antigens, thus inhibiting the immune system disorders and anti-inflammatory effects associated with HAdV infection. China’s national guidelines for adenovirus pneumonia in children endorse the administration of IVIG for the treatment of SAP. The recommended dosage is 1 g/(kg. d)- 2d, and usually, IVIG should be combined with additional antiviral drugs or other therapies to maximize its therapeutic efficacy.

While glucocorticoids do not possess antiviral effects, they are primarily administered in HAdV infection to reduce inflammation in children experiencing a hyperinflammatory response. However, the use of glucocorticoids in immunocompromised children may increase the rate of secondary infection and enhance the risk of respiratory failure. The effectiveness of glucocorticoids in the treatment of SAP remains unclear, with no standardized dosage protocol [97]. Therefore, further clinical studies are required to verify their safety and efficacy.

There is evidence that treatment with HAdV high valent neutralizing antibody plasma improves survival in children with SAP [98, 99]. This therapeutic approach has shown promising safety with specific therapeutic effects. However, this treatment strategy is only empiric and needs to be validated by extensive clinical trials involving large-scale samples.

T-cell immunomodulatory therapy has emerged as a new direction of recent research in oncology and infectious diseases. However, its application in SAP patients is still in the initial stage, with several problems that need to be solved, including small-scale, large individual differences, and safety issues. Notably, the infusion of familial CD45RA − t cells has been shown to be effective in improving cellular immunity in immunocompromised patients [100]. At the same time, an allogeneic T cell therapy called Posoleucel is also undergoing multicenter clinical trials for adenovirus treatment [101]. In addition, a promising treatment for critically ill children involves immunomodulation utilizing adoptive HAdV-specific T-cell transplantation therapy. This innovative approach involves purifying and isolating specific T cells from healthy individuals using cytokine capture technology, replicating them in vitro, and introducing them into the patient’s body to achieve the purpose of T cell expansion and immune repair [102]. However, this transplant therapy is expensive and its use is mainly limited to treating malignant tumors. The good news is that virus-specific T cell therapies for Epstein-Barr virus and adenovirus infections in children are already being studied in multicenter studies and have preliminary efficacy [103, 104].

### Adjuvant therapy

The initial adjuvant therapy for children with AVP primarily focuses on respiratory assistance. Oxygen therapy can alleviate hypoxemia resulting from lung infection and reduce the risk of disease progression requiring invasive ventilators. In advanced cases, children often experience respiratory failure, and mechanically assisted ventilation or artificial airway-assisted therapy can effectively improve oxygen levels, reduce ventilator effort, and reduce symptoms.

Adjuvant ECMO treatment is utilized as a reserved therapy when adjunctive mechanical ventilation is ineffective for children with respiratory failure or acute respiratory distress syndrome and SAP [105]. Although this adjuvant ECMO therapy can minimize lung injury caused by mechanical ventilation, children should be closely monitored for potentially serious complications, including hemorrhage or pneumothorax. Research has demonstrated that ECMO had a high survival rate (73.2%) among children with severe disease. However, complications such as bleeding and drug-resistant bacterial infections remain challenges that must be addressed in the adjuvant treatment of ECMO [106]. In children with HAdV infection, the underdeveloped trachea increases the secretion of alveolar epithelial tissue after infection. This results in difficult discharge of sputum, which subsequently affects the pulmonary ventilation and pulmonary ventilation function of children. The use of bronchoscopic interventional therapy in pediatric respiratory diseases is becoming more prevalent. This technique allows for direct visualization of specific situations of the lesion site in the lung, providing a more intuitive view of the extent of inflammation and effectively removing the blocked mucus plug [107]. Concurrently, alveolar lavage fluid can be analyzed for pathogens to help in confirming the diagnosis and identifying potential co-infections [108]. Also, drugs can be administered locally to increase the effective concentration of the drug and achieve improved therapeutic outcomes. Flexible bronchoscopy adjuvant therapy enables more accurate etiological detection and targeted drug delivery for children, ultimately resulting in more effective treatment effects [109].

### Co-infection treatment

Individuals with HAdV infection are more susceptible to additional bacterial or viral infections, which can greatly exacerbate the systemic inflammatory response in children. Influenza and respiratory syncytial virus are commonly observed as co-infected viruses, while Streptococcus pneumoniae and Haemophilus influenzae are common co-infected bacteria [110, 111]. Additionally, these patients are susceptible to mycoplasma infection. Doctors can quickly detect respiratory pathogens using comprehensive screening methods, including blood, sputum, and alveolar lavage fluid cultures, along with analyzing abnormalities in laboratory test markers such as inflammatory factors. For mixed viral infections, treatment mainly uses pathogen-specific antiviral drugs, such as oseltamivir, for influenza virus infection. However, clinical pharmacists must be consulted regarding the potential risks of pediatric use. Broad-spectrum antibiotics such as third-generation cephalosporins or carbapenems should be used prior to identifying the specific pathogen responsible for co-bacterial infection. Once the diagnostic results of the bacterial culture are available, treatment should be promptly adjusted for targeted therapy to avoid the development of drug resistance. Additionally, when selecting medications for children, it is important to consider their individual drug tolerance and ensure appropriate dosing.

Children with severe pneumonia are susceptible to co-infection during treatment, such as mechanically assisted breathing or ECMO. When glucocorticoid treatment is indicated, it must be administered alongside effective anti-infective drugs to avoid aggravation of infection and reduce prognosis.

To conclude, AVP in children is usually mild and resolves on its own. However, if left untreated without intervention, it can progress to SAP with a high mortality rate and is associated with various extrapulmonary and extrapulmonary complications affecting multi-system and multi-organs. HAdV has multiple serotypes, and the genetic diversity between each serotype contributes to its high variability. Furthermore, the recombination of HAdV with DNA from other viruses or host cells results in the emergence of new biological properties. Consequently, progress in developing specific drugs and new treatments for HAdV has been limited, with no specific treatment currently available. Clinicians must employ a combination of antiviral therapy, respiratory support, and immunomodulation and closely monitor them to avoid co-infections and extrapulmonary and extrapulmonary comorbidities [112]. The early diagnosis of SAP in children remains challenging, and many studies have been devoted to finding the early predictive factors and establishing reliable clinical prediction models through various comprehensive indicators. These efforts aim to assist clinicians in identifying SAP at an early stage.

## Conclusions

The pathogenic mechanism of AVP in children is complex. Once the virus invades respiratory epithelial cells, it initiates an innate immune response and releases numerous inflammatory mediators. The resulting cellular damage and inflammatory responses lead to severe lung infection. A hyperactive immune system generates a “cytokine storm” that exacerbates lung tissue damage. Immunocompromised children have impaired numbers and functionality of immune cells, which affects the antibody production by B cells and the T cell’s immune response to HAdV. The compromised cellular immune function has a limited antiviral effect, increasing the progress to severe HAdV infection. While numerous studies have explored the etiology, function, and pathogenicity of HAdV, the exact mechanism of the specific pathogenicity of different adenovirus serotypes warrants additional investigation. Currently, there are no specific antiviral drugs for treating AVP in children. However, potential future treatment directions may involve the development of new broad-spectrum antiviral drugs and immunomodulatory therapies to enhance the immune response in children. In any case, it is crucial to identify SAP in children as early as possible to avoid serious complications and actively improve prognosis and reduce sequelae.

## Data Availability

Not applicable.

## Abbreviations

HAdV: Human adenovirus
AVP: Adenovirus pneumonia
SAP: Severe adenovirus pneumonia
ARTI: Acute respiratory tract infection
VAMPs: Virion-associated molecular patterns
pDCs: Plasmacytoid dendritic cells
cGAS: Circular GMP-AMP synthetase
cGAMP: Circular GMP-AMP
MAF: Macrophage activating factor
MBL: Mannose-binding lectin
CK: Cytokines
APCs: Antigen-presenting cell
IL: Interleukins
TNF: Tumor necrosis factor
IFN: Interferon
TRIM21: Tripartite motif containing 21
Tregs: Regulatory T cells
PFN: Perforin
GZM: Granzyme
VEGFR-2: Vascular endothelial growth factor receptor 2
IVIG: Intravenous immunoglobulin
ECMO: Extracorporeal membrane oxygenation
